# Association between intra-articular hyaluronic acid injections in delaying total knee arthroplasty and safety evaluation in primary knee osteoarthritis: analysis based on Health Insurance Review and Assessment Service (HIRA) claim database in Republic of Korea

**DOI:** 10.1186/s12891-024-07698-2

**Published:** 2024-09-04

**Authors:** Jun-Gu Park, Juho Sim, Seung-Beom Han

**Affiliations:** 1 Department of Orthopaedic Surgery, College of Medicine, Anam Hospital, Korea University, 73, Goryeodae-ro, Seongbuk-gu, Seoul, South Korea; 2 Department of Preventive Medicine, Yonsei University College of Medicine, Seoul, South Korea

**Keywords:** Intra-articular hyaluronic acid, Total knee arthroplasty, Knee osteoarthritis, Corticosteroid injection

## Abstract

**Background:**

The prevalence of knee osteoarthritis (KOA), a progressive degenerative disease, is gradually increasing, and it is a progressive degenerative disease. In patients with mild-to-moderate KOA, intra-articular hyaluronic acid (IA-HA) has been shown to be an effective non-operative treatment option that can provide significant pain relief and symptom improvement by increasing intra-articular viscoelasticity. This study aimed to evaluate the efficacy of IA-HA injections in delaying total knee arthroplasty (TKA) and the safety of IA-HA according to IA-HA type and combination with intra-articular corticosteroid (IA-CS) using a large health insurance claim database.

**Methods:**

For this retrospective cohort study, the study population included patients aged ≥ 50 years with a first diagnosis of KOA between 2009 and 2014, who underwent TKA by 2020, using the Health Insurance Review and Assessment Service claim database in Republic of Korea. IA-HA injections were categorized as single or multiple injection regimen agents. Cox proportional hazard models estimated hazard ratios (HR) for TKA risk, adjusted for covariates. Logistic regression assessed the occurrence of adverse events after IA-HA administration.

**Results:**

In all, 36,983 patients were included. Patients who received IA-HA injections had a significantly longer time to TKA compared to those who did not (mean delay of approximately 1 year). The IA-HA group had a significantly lower risk of TKA (HR: 0.61, 95% CI: 0.60–0.62) than non-IA-HA group after adjusting for covariates, which included age, sex, medical history, number of hospital beds, and CS injection. Single injection IA-HA regimen agents showed the longest time to TKA and lowest risk (HR: 0.56, 95% CI: 0.53–0.59). TKA risk decreased with the number of IA-HA cycles. Adverse events occurred in 6.7% of IA-HA cases without CS, with very low incidence of infection. Multiple injection regimen agents (multiple injection regimen 7.0% vs. single injection regimen 3.6%) and concurrent IA-CS use (concurrent IA-CS use 13.9% vs. IA-HA only 6.7%) were associated with higher infection risk.

**Conclusion:**

IA-HA injections were associated with a significant delay in TKA among patients with KOA. Single-injection regimen agents had the lowest TKA risk. Infection risk increased with multiple injections and concurrent IA-CS use. These findings could suggest the use of IA-HA as an effective non-operative intervention option for managing KOA and delaying TKA. Careful selection of IA-HA type and consideration of concurrent IA-CS use could play a role in delaying the time to TKA and reducing complications.

**Supplementary Information:**

The online version contains supplementary material available at 10.1186/s12891-024-07698-2.

## Introduction

Knee osteoarthritis (KOA) is a progressive degenerative disease characterized by cartilage degradation and joint destruction, resulting in pain, functional impairment, and disability [1, 2]. It accounts for a substantial portion of all osteoarthritis cases [3] and the prevalence is gradually increasing with increasing life expectancy and subsequent emergence of an aging society. From 1990 to 2019, there was a consistent global annual increase in KOA of 3.2%, highlighting its significance as a major public health concern [3]. Furthermore, by 2030, Republic of Korea is expected to have the highest global life expectancy, reaching 86.7 years [4], further emphasizing KOA as a major public health concern.

Knee osteoarthritis manifests with a progression of disease severity [5–7]. Non-surgical treatments are typically employed in the early stages, while a transition to pharmacological interventions is made to manage symptoms as the disease progresses. Surgical treatments, such as arthroplasty, may be considered for advanced KOA cases. There has been an increasing trend in the incidence of total knee arthroplasties in the past decades worldwide [8–10]. While total knee arthroplasty (TKA) demonstrates satisfactory long-term survival rates, inherent risks, including infection, periprosthetic fracture, and implant loosening, pose potential lifelong concerns [11, 12]. Therefore, delaying surgical treatment through effective and appropriate non-operative interventions is crucial from a patient and socioeconomic perspective.

Intra-articular hyaluronic acid (IA-HA) has shown effectiveness as a non-operative treatment option in providing significant pain relief and symptom improvement by increasing intra-articular viscoelasticity in patients with mild-to-moderate KOA [13, 14]. The IA-HA has also demonstrated a favorable safety profile for both short-term and long-term use [15–17]. However, the current guidelines for KOA management exhibit inconsistent recommendations for IA-HA utilization [5–7]. These discrepancies can be attributed to the lack of standardized treatment protocols and the variety of IA-HA products, which differ in terms of molecular HA weight, cross-linking methods, HA sources, the number of injections per treatment course, and treatment cycles [13, 14].

Recent systematic reviews conducted on IA-HA, specifically comparing the efficacy of multiple versus single injection regimens, have shown inconsistent results [18, 19]. One study reported that a multiple injection regimen of IA-HA provided pain improvement, while another found that a single-injection regimen had similar efficacy, potentially offering better cost-effectiveness and convenience. Therefore, further study is needed to compare their effectiveness in reducing the risk of arthroplasty.

Furthermore, IA-HA injections are often administered concurrently with intra-articular corticosteroid (IA-CS) injections to provide short-term symptom relief through the immediate anti-inflammatory effects of CS. However, this combination approach hinders the accurate assessment of IA-HA’s independent efficacy. Previous large cohort studies have used claims databases to demonstrate the effectiveness of IA-HA in delaying the incidence for TKA [20, 21]. However, they did not adequately address the limitations associated with various IA-HA formulations and the concurrent use of IA-CS. Moreover, concerns arise regarding the potential for increased infection rates and adverse events with multiple IA-HA cycles and concurrent IA-CS use.

In the Republic of Korea, national insurance provides coverage for IA-HA injections in patients diagnosed with mild to moderate knee joint osteoarthritis (Kellgren-Lawrence grade I, II, III) based on radiographic examination. For TKA, insurance coverage is available for cases where conservative therapy fails to alleviate symptoms (such as pain and functional impairment) for more than 3 months. The Health Insurance Review and Assessment (HIRA) data are collected during the process of reimbursing healthcare provider, which provides a comprehensive and reliable source of information [22] on the management and treatment of knee osteoarthritis in routine clinical practice in the Republic of Korea.

Therefore, given its ability to capture recent trends, this study aimed to evaluate the efficacy of IA-HA that contributes to delaying TKA in KOA and the safety of IA-HA according to IA-HA agent type and combination of IA-CS using the Health Insurance Review and Assessment (HIRA) Service’s healthcare database of the Korean population.

## Materials and methods

### Study Population and study design

This retrospective cohort study used health insurance claims data from HIRA in Republic of Korea. The claims data include basic demographics, hospital visit records, procedure, and drug prescription records of all Korean citizens. The initial inclusion criteria of this study were as follows: (1) participants aged ≥ 50 years, (2) not previously diagnosed with KOA between January 2008 and February 2009, (3) first KOA diagnosis (ICD-10 M17) at hospitalization or outpatient visit was between March 2009 and February 2014, and (4) underwent TKA between March 2014 and December 2020, as identified by the procedural codes for TKA (N2072, N2077). This study was approved by the institutional review board (Public Institutional Review Board Designated by Ministry of Health and Welfare: P01-202108-21-003).

**Fig. 1:**
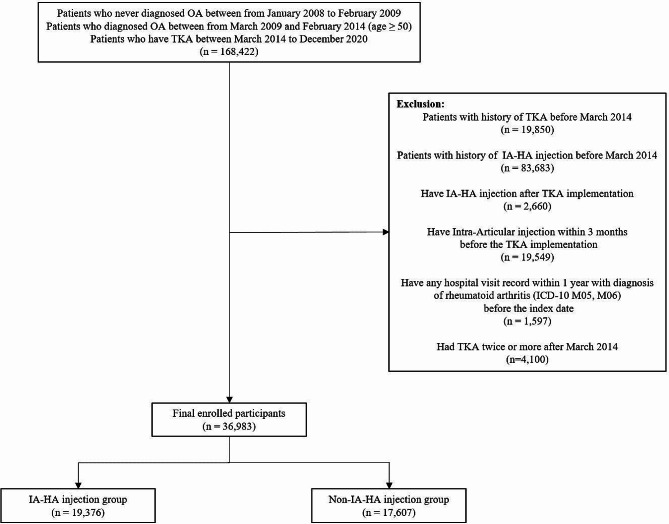
The schematic flow of the inclusion and exclusion criteria of participants

Out of 168,422 enrolled patients, 36,983 were eventually included after applying exclusion criteria, which included prior TKA or IA-HA injection, recent intra-articular injection, rheumatoid arthritis diagnosis, and multiple TKAs (Fig. 1).

A retrospective analysis was undertaken to calculate the survival time to TKA among patients with KOA. The survival time of each individual was measured from the time of the first KOA diagnosis (index date) until the TKA event. The safety of HA injection was evaluated with the occurrence of infection after injection. An adverse event was defined as a visit to the hospital within 2 weeks after HA (*n* = 19,376) or CS (*n* = 11,910) administration, via an outpatient clinic limited to the claims collected from orthopaedics and related departments, with procedure codes associated with suspected infection. Arthroscopy and procedures in knee joint such as knee joint aspiration (symptoms suggestive of infection; pain, swelling, erythema) performed within 2 weeks after injection was defined as an adverse event.

### Variables

IA-HA injection was defined according to the procedural code records of all intra-articular injections with HA in the HIRA database. A single injection regimen was defined based on the use of 1,4-butanediol diglycidyl ether cross-linked HA (main substance code assigned by Korean Ministry of Food and Drug Safety: 526001BIJ, 526030BIJ), whereas a multiple injection regimen was defined based on the use of general HA (530045BIJ, 229228BIJ).

The covariates used in this study were age, sex, medical history, and number of hospital beds. Medical history included hypertension, diabetes mellitus, intracranial hemorrhage, ischemic stroke, atrial fibrillation, liver cirrhosis, pulmonary embolism, deep vein thrombosis, angina, end-stage renal disease, chronic kidney disease, dyslipidemia, heart failure, peripheral arterial disease, chronic obstructive pulmonary disease, cancer, and metastatic cancer. The number of hospital beds was stratified into four groups: <30, 30–99, 100–299, and ≥ 300.

### Data analyses

Baseline characteristics of all participants stratified by IA-HA injection were presented as frequency (%) using *chi*-square test or mean (standard deviation) using analysis of variance (ANOVA) test. We estimated the hazard ratios (HR) and 95% confidence intervals (CI) of the outcomes using Cox proportional hazard models. The follow-up period was defined as the period from the index date to primary TKA. The multivariate model was adjusted for all covariates including age, sex, medical history, number of hospital beds, and CS injection. The HR of the outcomes was calculated in the IA-HA injection group and compared to that of the non-IA-HA injection group. The efficacy of multiple versus single injection regimens, as well as the combined effect of IA-HA and CS injection was tested using multivariate Cox proportional hazards models. The survival rate in terms of TKA conversion was assessed and compared using Kaplan–Meier survival curve and log-rank test.

Additionally, the overall incidences of adverse events according to IA-HA or CS treatment group were assessed. Logistic regression was performed to compare the occurrence of adverse events between the HA treatment group with and without IA-CS, considering the duration of treatment and the number of administrations, and to calculate the corresponding odds ratios (ORs). All statistical analyses were performed using SAS Enterprise Guide version 9.4.2 (SAS Institute, Cary, NC), and a *P*-value < 0.05 was regarded as statistically significant.

## Results

The demographic and clinical information of the study population are presented in Table 1, including patients who received IA-HA injections (*n* = 19,376) and those who did not (*n* = 17,607). The variables included were age, sex, presence of various medical conditions, number of hospital beds, concurrent CS injection, and number of IA-HA cycles. The mean patient age was 64.5 years, with a higher proportion of women (81.34%) than men (18.66%). The proportions of women were similar between the IA-HA and non-IA-HA groups and the difference was not statistically significant (*P* = 0.053). Compared with the IA-HA injection group, the non-IA-HA injection group showed a significantly higher proportion of patients with hypertension (43.00 vs. 44.53%, *P* = 0.003) and diabetes (11.99 vs. 13.44%, *P* < 0.001). The 79.77% of the patients visited hospitals with fewer than 30 beds. The IA-HA injection group had a significantly higher proportion of patients visiting/admitted to hospitals with a smaller number of beds. Furthermore, the proportion of patients who underwent CS injection was lower than that of the non-IA-HA injection group (38.92 vs. 67.64%, *P* < 0.001). Among those who received IA-HA injections, the mean number of IA-HA cycles was 2.09, with the majority receiving one or two injections. The number of patients who received concomitant medications, which included non-steroidal anti-inflammatory drugs (NSAIDs), analgesics, NSAIDs and analgesics, oral steroids, and intra-articular injection using tissue restorative biomaterials containing polynucleotides were similar between IA-HA injection group and the non-IA-HA injection group.

**Table 1:** Baseline characteristics of participants stratified by intra-articular injection of hyaluronic acid injection

			IA-HA injection		*P*-value***
		Total (*n* = 36,983)	Injection (*n* = 19,376)	Non- injection (*n* = 17,607)	
**Age(years)**,** median (IQR)**		**65 (60–69)**	**64 (59–69)**	**66 (61–70)**	**< 0.001**
**Sex**,** n (%)**					0.053
Men		6,901 (18.66)	3,688 (19.03)	3,213 (18.25)	
Women		30,082 (81.34)	15,688 (80.97)	14,394 (81.75)	
**Medical History**,** n(%)**					
**Hypertension**		**16**,**173 (43.73)**	**8**,**332 (43.00)**	**7**,**841 (44.53)**	**0.003**
**Diabetes mellitus**		**4**,**690 (12.68)**	**2**,**324 (11.99)**	**2**,**366 (13.44)**	**< 0.001**
Intracranial hemorrhage		363 (0.98)	189 (0.98)	174 (0.99)	0.901
**Ischemic stroke**		**1**,**188 (3.21)**	**574 (2.96)**	**614 (3.49)**	**0.004**
Atrial fibrillation		139 (0.38)	81 (0.42)	58 (0.33)	0.164
Liver cirrhosis		108 (0.29)	62 (0.32)	46 (0.26)	0.296
Pulmonary embolism		6 (0.02)	4 (0.02)	2 (0.01)	0.484
Deep vein thrombosis		24 (0.06)	13 (0.07)	11 (0.06)	0.862
Angina		2,139 (5.78)	1,125 (5.81)	1,014 (5.76)	0.846
End stage renal disease		40 (0.11)	15 (0.08)	25 (0.14)	0.059
Chronic kidney disease		124 (0.34)	59 (0.30)	65 (0.37)	0.283
**Dyslipidemia**		**7**,**748 (20.95)**	**4**,**304 (22.21)**	**3**,**444 (19.56)**	**< 0.001**
Heart failure		416 (1.12)	218 (1.13)	198 (1.12)	0.996
Peripheral arterial disease		3,149 (8.51)	1,671 (8.62)	1,478 (8.39)	0.429
Chronic obstructive pulmonary disease		2,606 (7.05)	1356 (7.00)	1,250 (7.10)	0.704
Cancer		1,415 (3.83)	725 (3.74)	690 (3.92)	0.375
**Metastatic cancer**		55 (0.15)	22 (0.11)	33 (0.19)	0.066
Number of Hospital Bed*,** n (%)**					**< 0.001**
≥ 300		2,075 (5.61)	930 (4.80)	1,145 (6.50)	
300 − 100		3,099 (8.38)	1,453 (7.50)	1,646 (9.35)	
100 − 30		2,306 (6.24)	1,090 (5.62)	1,216 (6.91)	
< 30		29,503 (79.77)	15,903 (82.08)	13,600 (77.24)	
**CS Injection**, n(%)**					
Yes		**19**,**451 (52.59)**	**7**,**541 (38.92)**	**11**,**910 (67.64)**	**< 0.001**
**Period from knee OA diagnosis to TKA (days)**,** median (IQR)**		**2542 (1922–3147)**	**2734 (2168-3291.5)**	**2307 (1694–2937)**	**< 0.001**
**Number of IA-HA injections**,** mean** ± **SD**		2.09 ± 1.60	2.09 ± 1.60		
**Number of IA-HA injections**,** n(%)**					
1		9,936 (26.87)	9,936 (51.28)		
2		4,228 (11.43)	4,228 (21.82)		
3		2,306 (6.24)	2,306 (11.90)		
4		1,264 (3.42)	1,264 (6.52)		
≥ 5		1,642 (4.44)	1,642 (8.47)		

* Arranged by institution where osteoarthritis was first diagnosed

** Prescribed concomitant corticosteroid at least once in the time period (define combined as having a record of both intra-articular hyaluronic acid injection and corticosteroid injection within 1 week or having a record of prescribed corticosteroid in non-intra-articular hyaluronic acid injection)

*** Continuous variables: Analysis of Variance (ANOVA, Levene’s equal variance test), categorical variables: *Chi*-square test

Values are expressed as frequencies (%) or medians (Interquartile range), as appropriate

IQR, Interquartile Range; IA-HA, Intra-articular Hyaluronic acid; OA, osteoarthritis; TKA, total knee arthroplasty; CS, Corticosteroid; SD, standard deviation

**Fig. 2:**
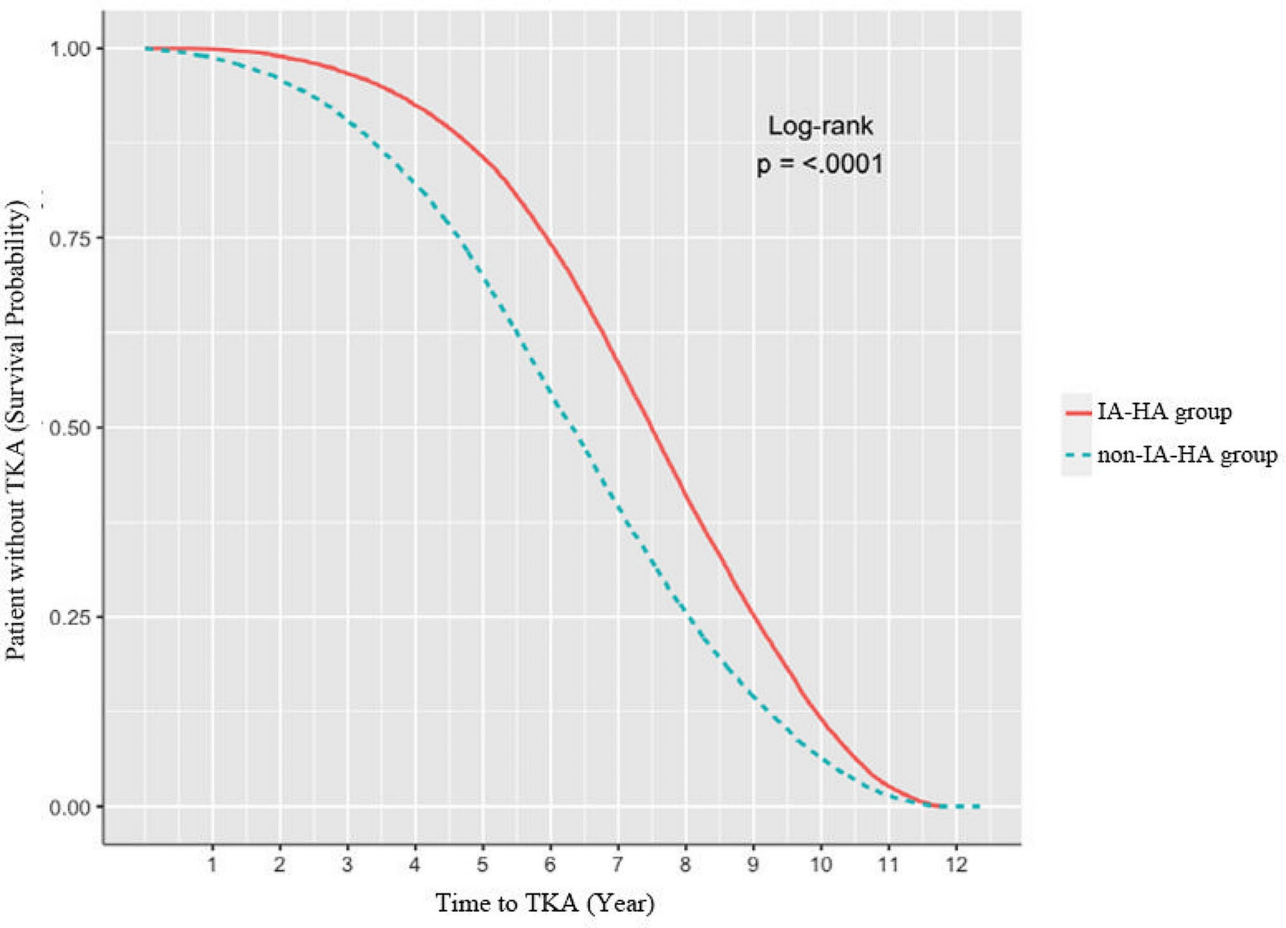
Kaplan-Meier curves and Log-rank test plot of intra-articular hyaluronic acid injection

The IA-HA injection group showed a significantly longer time to TKA than the non-IA-HA injection group (2,690.8 ± 790.9 days vs. 2,296.7 ± 877.2 days; *P* < 0.001), indicating approximately a 1-year delay in TKA in the IA-HA injection group (Fig. 2). The crude HR (95% CI) of TKA in the IA-HA injection group was 0.69 (0.68–0.71), compared to that in the non-IA-HA injection group (Table 2). The result was not attenuated even after controlling for all covariates including age, sex, number of hospital beds, presence of medical histories, and CS injection [multivariable model: adjusted HR (95% CI) 0.61 (0.60–0.62)].

**Table 2:** The risk of total knee arthroplasty according to intra-articular hyaluronic acid injection, corticosteroid, regimens, or number of intra-articular hyaluronic acid cycle

Variables	Number of participants	HR (95% CI)	
		Univariate	Multivariate model1*
**IA-HA**			
Non-injections	17,607	1.00 (reference)	1.00 (reference)
Injections	19,376	0.69 (0.68–0.71)	0.61 (0.60–0.62)
**Regimens of IA-HA injections**			
Non-injections	17,607	1.00 (reference)	1.00 (reference)
Single injection regimens	1,583	0.62 (0.59–0.66)	0.56 (0.53–0.59)
Multiple injection regimens	17,793	0.7 (0.68–0.71)	0.62 (0.6–0.63)
**Number of IA-HA cycle**			
Non-injections	17,607	1.00 (reference)	1.00 (reference)
Cycle 1	9,936	0.8 (0.78–0.82)	0.67 (0.65–0.69)
Cycle 2	4,228	0.7 (0.68–0.73)	0.64 (0.62–0.66)
Cycle 3	2,306	0.59 (0.57–0.62)	0.55 (0.53–0.58)
Cycle 4	1,264	0.55 (0.51–0.58)	0.52 (0.49–0.55)
≥ Cycle 5	1,642	0.49 (0.46–0.51)	0.49 (0.46–0.51)
**Corticosteroid**			
Non-injections	17,532	1.00 (reference)	1.00 (reference)
Injections	19,451	0.83 (0.81–0.85)	0.67 (0.66–0.69)

*Adjusted for age, sex, medical history, hospital size (number of hospital bed), and corticosteroid injection

IA-HA, Intra-articular Hyaluronic acid; CS, Corticosteroid

**Fig. 3:**
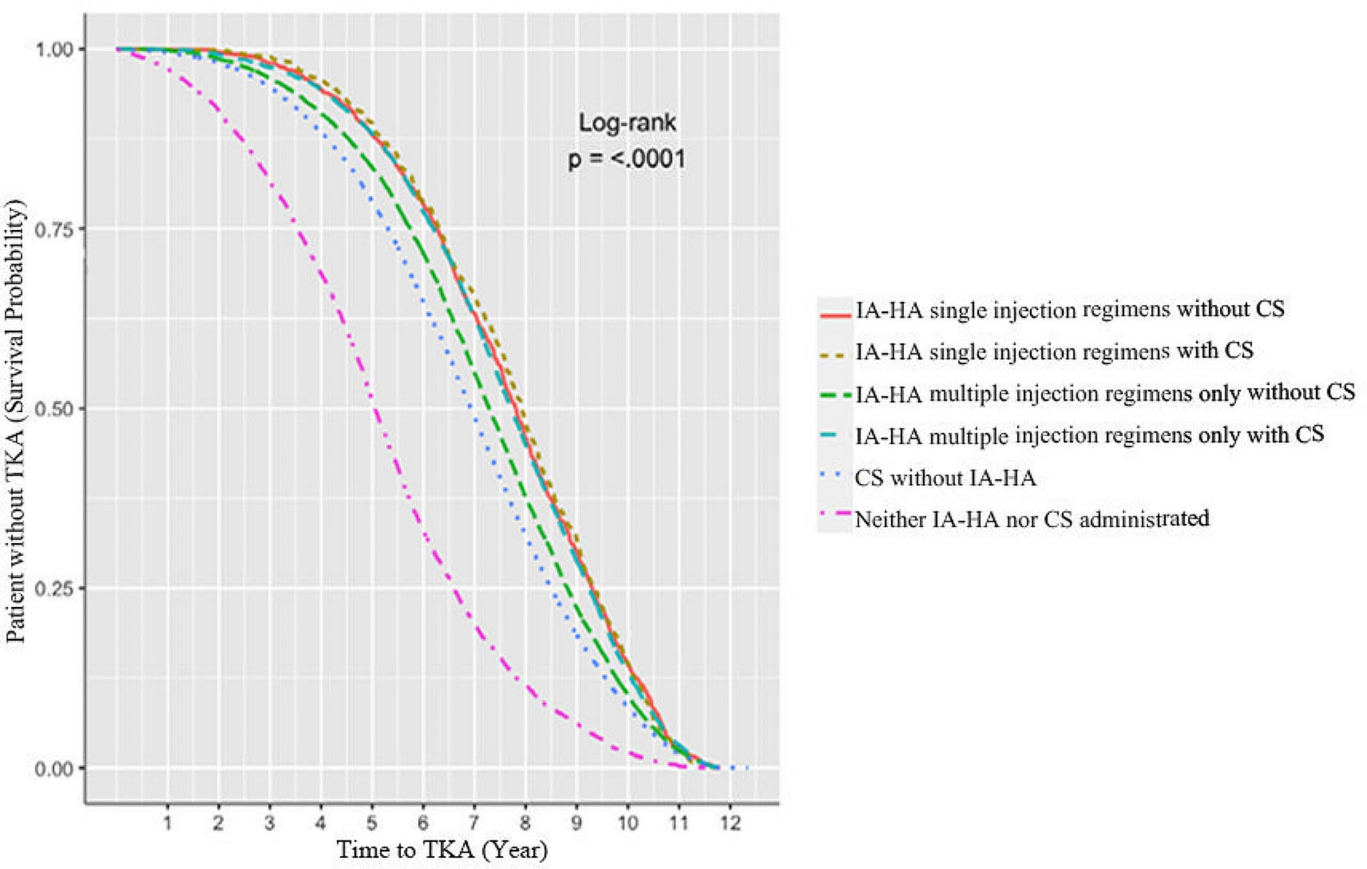
Kaplan-Meier curves and Log-rank test plot of intra-articular hyaluronic acid injection regimen and corticosteroid

**Fig. 4:**
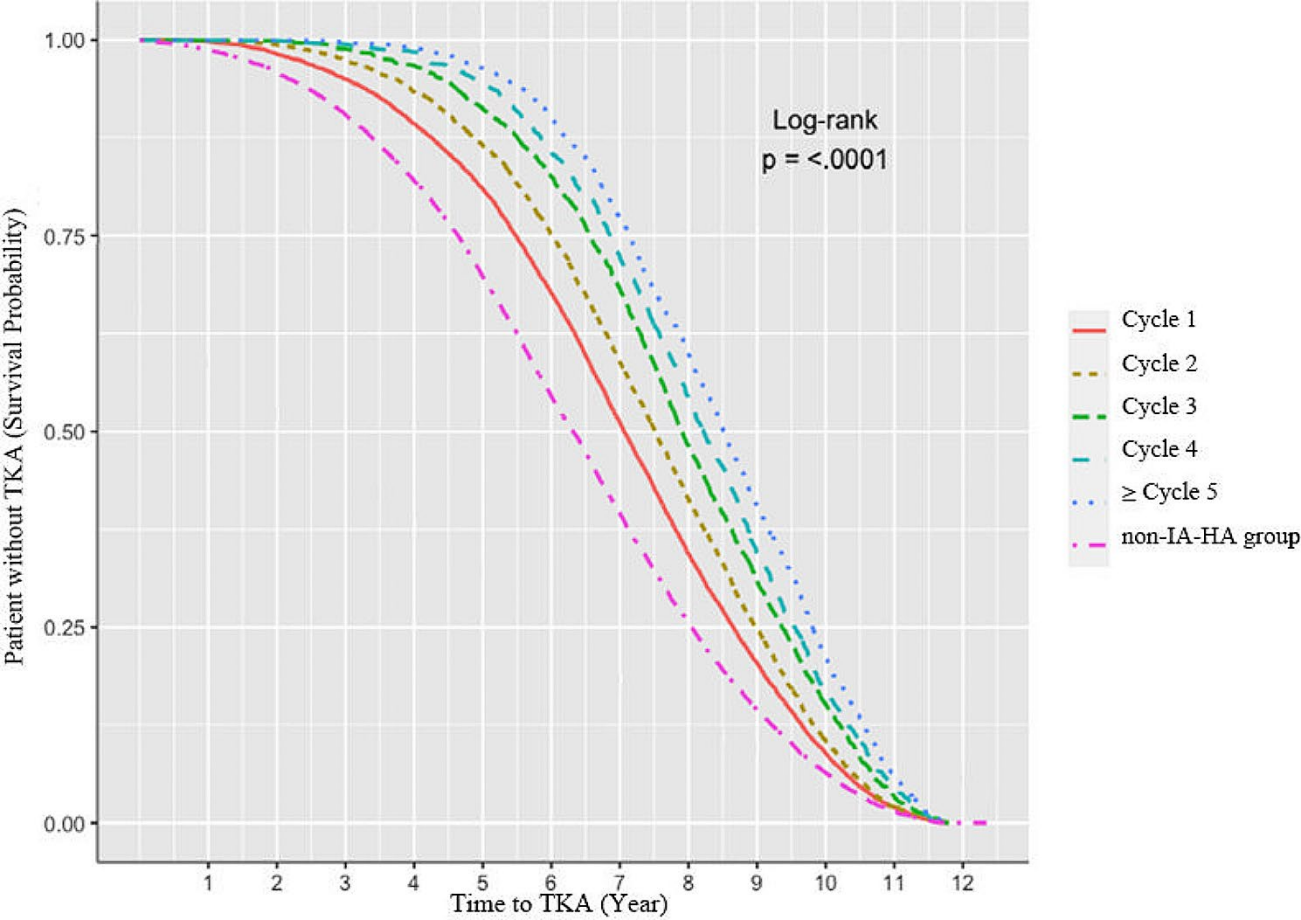
Kaplan-Meier curves and Log-rank test plot of number of intra-articular hyaluronic acid cycle

When comparing the time to TKA according to the type of HA agent, the single injection IA-HA regimen group showed the longest time to TKA of 2,809.6 ± 753.0 days, followed by 2,680.2 ± 793.3 days in the multiple injection regimen group and 2,296.7 ± 877.2 days in the non-IA-HA group (Fig. 3). Compared to the non-IA-HA injection group, the single and multiple IA-HA injection regimen groups had a lower TKA risk [adjusted HR 0.56 (95% CI, 0.53–0.59) and 0.62 (0.60–0.63), respectively, *P* < 0.001; Table 2)], after adjusting for covariables. Additionally, the association between number of IA-HA injection cycles and TKA risk had a dose-dependent relationship, with the HR ranging from 0.67 for one cycle to 0.49 for more than five cycles, compared to the non-IA-HA injection group (Fig. 4).

Table 3 indicates HR (95% CI) for TKA risk in different treatment groups. Patients who received single injection regimens regardless of CS injection had a significantly lower TKA risk compared to those who received neither IA-HA nor CS injections, with adjusted HR (95% CI) of without/with CS injections as 0.37 (0.35–0.40) and 0.37 (0.34–0.40), respectively.

**Table 3:** The risk of total knee arthroplasty according to the intra-articular hyaluronic acid injection regimen and corticosteroid

			Hazard Ratio (95% Confidence interval)	
	Number of participants	Period from knee OA diagnosis to TKA (days)	Univariate	Multivariate model*
Neither **IA-HA** nor **CS** administrated	5,697	1845 (1293–2411)	1.00 (reference)	1.00 (reference)
**IA-HA** single injection regimens without **CS**	884	2854 (2290–3395.5)	0.36 (0.33–0.38)	0.37 (0.35–0.40)
**IA-HA** single injection regimens with **CS**	699	2894 (2303–3413)	0.35 (0.33–0.38)	0.37 (0.34–0.40)
**IA-HA** multiple injection regimens only without **CS**	10,951	2660 (2095–3218)	0.42 (0.41–0.44)	0.44 (0.43–0.45)
**IA-HA** multiple injection regimens only with **CS**	6,842	2814 (2263–3375)	0.36 (0.35–0.38)	0.38 (0.37–0.40)
**CS** without **IA-HA**	11,910	2530 (1939–3100)	0.47 (0.46–0.49)	0.48 (0.47–0.5)

*Adjusted for age, sex, medical history, hospital size (number of hospital bed)

IA-HA, Intra-articular Hyaluronic acid; CS, Corticosteroid

In Supplementary Table 1, the incidence of adverse events within 2 weeks after IA-HA administration without concurrent CS use was 6.7%, and there was no arthroscopy performed for suspected infection in the IA-HA injection without CS group. The low rate of arthroscopy was observed in the combined IA-HA with CS injection group (1 case, 0.01%), followed by the CS without IA-HA injection group (5 cases, 0.04%). Based on the logistic regression analysis (Table 4), compared to those with IA-HA injection, patients with concurrent use of IA-CS was associated with a higher risk of infection (adjusted OR [CI]: 1.80 [1.62–1.99]). The number of injection cycles were higher and duration of injection periods were longer in the patients who reported suspected infection compared to those who did not report the suspected infection.

**Table 4:** Logistic regression between intra-articular hyaluronic acid injection related information and adverse events including procedures associated with suspected infection in patients with intra-articular hyaluronic acid injection (*N* = 19, 376)

	Univariate		Multivariate model*	
	OR	(95% CI)	OR	(95% CI)
**Treatment Group**				
**IA-HA** injection regimens without **CS**	ref.			
**IA-HA** injection regimens with **CS**	2.242	(2.037–2.468)	1.795	(1.622–1.986)
Number of IA-HA Injections (cycles)	1.313	(1.283–1.344)	1.220	(1.169–1.272)
Total duration of IA-HA injection (years)	1.343	(1.304–1.383)	1.060	(1.005–1.119)

OR, Odds Ratio, CI, Confidence Interval, IA-HA, Intra-articular Hyaluronic acid; CS, Corticosteroid

*Adjusted for Number of intra-articular hyaluronic acid Injections, Total duration of intra-articular hyaluronic acid injection

## Discussion

The IA-HA is used in patients with primary osteoarthritis as a concept of viscosupplementation to enhance lubrication and viscoelasticity in the knee joint. Various types of HA agents are available worldwide for KOA treatment, with varying molecular weights, HA concentration, injection volume, duration of treatment courses, and number of injections per course [14]. Recent research has indicated that high molecular weight (HMW) HA has higher affinity compared to low molecular weight (LHW) HA [13]. Furthermore, cross-linked HMW HA agents have been introduced to prolong the intra-articular residence time of HMW HA [16]. Consequently, the efficacy of HA agents can vary significantly among different formulations. The IA-HA not only enhances joint lubrication but also reduces inflammation in KOA by binding to CD44 receptors, lowering interleukin-1B expression, and decreasing matrix metalloproteinase (MMP) production, which helps protect cartilage from degradation.

Similarly, previous cohort studies demonstrated that IA-HA treatment delays the incidence for TKA in KOA. In a study analyzing the time to TKA in 744,734 patients with KOA using an administrative claims database, the TKA-free survival rates for the IA-HA treatment group were 85.8 and 70% at one and two years, respectively, which were higher than the rates of 74.1 and 63.7% at one and two years, respectively, for the non-IA-HA group [19]. Another study analyzing patients who underwent TKA after a diagnosis of KOA, with a total of 182,022 patients, demonstrated an average delay of 332 days. Specifically, while the non-IA-HA group exhibited a mean time to TKA of 270 days, this duration extended to 602 days for those in the IA-HA group. Furthermore, the delay effect was more pronounced with an increased number of HA treatment cycles [20]. Recent systematic reviews also reported a delay effect to TKA of approximately 9.8 months with IA-HA [23]. The present study revealed that the mean time to TKA after KOA diagnosis was 7.4 and 6.3 years in the HA group and non-IA-HA groups, respectively, suggesting that patients receiving HA treatment experienced an approximate 1-year delay until TKA compared to those without HA treatment. When comparing the time to TKA according to HA agent type, the single injection regimen group showed a longer time to TKA of 7.7 years, while the multiple injection regimen group and the non-IA-HA group had times to TKA of 7.3 and 6.3 years, respectively. The interaction analysis of IA-HA and CS injection showed that TKA risk in the single injection regimen was significantly lower than in the multiple regimen and non-IA-HA groups, regardless of concurrent CS injection.

Regarding adverse events, the overall incidence of injection-related complications, such as pain, warmth, redness, and swelling, is 3–6%. Acute septic arthritis is one of the most concerning and potentially fatal complications associated with IA-HA injections, with incidence rates ranging from 0.001 to 0.072% [24]. In this study, the overall incidence of adverse events in the total HA administration without CS group was 6.7%, which did not significantly differ from previously reported incidences. The incidence rate of arthroscopy for suspected infection was 0%, but it increased to 0.01 and 0.04% in cases where CS in combination with IA-HA injection or CS was administered alone, respectively. Furthermore, the incidence of infection tended to increase with an increasing number of HA administrations. These findings suggest that multiple HA injection agents increase the infection risk compared to crosslinked-single HA agents. Additionally, the group receiving combined CS injections had a higher risk of infection compared to the group without CS.

One of the strengths of our study was the use of a large nationwide health insurance claims database, which enabled analysis of a large sample of patients with KOA. This large sample size improved the generalizability of our results, especially for the Asian population. Additionally, we have included several covariates that could potentially impact TKA risk, including the sex, considering that it is well-known that KOA is more prevalent in women than in men worldwide, including in the Republic of Korea [9, 25]. With strict reimbursement guidelines for IA-HA injections for patients with KOA, the results might reflect the clinical practice of IA-HA injection in Republic of Korea. Our study also showed that the use of IA-HA injections was associated with a lower HR of TKA even after controlling for various medical histories. Furthermore, the current study provides valuable information regarding the use of IA-HA injections in delaying TKA in the Asian population.

The efficacy of IA-HA can be determined in several ways including pain relief, delaying surgery, reduction in medication use, earlier return to work, and improved quality of life. Delaying TKA is considered the most stringent and practical indicator to evaluate patient outcomes and cost-effectiveness for policymakers. Previous studies, including the current study, have consistently reported a delay effect of approximately one year, and the results are predominantly based on patients who underwent TKA [19, 20]. Therefore, the actual delay effect is expected to be even greater than reported. These findings support the use of IA-HA injections as a viable alternative for managing the symptoms of knee osteoarthritis and delaying the surgical treatment. Furthermore, this study emphasizes the importance of selecting the optimal HA product to maximize the likelihood of delaying the time to TKA for patients with KOA.

However, our study has several limitations. This was an observational study using the claims data collected only in the Republic of Korea; therefore, we could not establish a causal relationship between IA-HA injections and TKA risk. Moreover, the use of claims data might not reflect unmeasured confounders, such as BMI, activity level, radiographic severity of KOA, and outcomes of adverse events. These factors could potentially confound the association between IA-HA injections and TKA risk. Additionally, this study focused on individuals who underwent TKA during the study period, leading to right-truncated survival data. Excluding patients who did not undergo TKA may have resulted in biased estimations of the association between the initiating event (KOA diagnosis) and the event of interest (TKA) [26]. Inverse probability weighting and expanding the study population to include both individuals who underwent TKA and those who did not may assist in better understanding of the association between KOA diagnosis and TKA [27].

## Conclusion

This study demonstrated that IA-HA injections can significantly delay the time to TKA in KOA patients. The delay in TKA was influenced by the type of IA-HA used, with cross-linked single injection regimens showing favorable results in terms of TKA delay. The risk of TKA is also influenced by the number of IA-HA cycles, showing a dose-dependent relationship. The incidence of adverse events was generally low, but multiple injections and corticosteroid use increase infection risk. Furthermore, this study emphasizes the importance of selecting the optimal HA product to maximize the likelihood of delaying the time to TKA for patients with KOA.

## Electronic supplementary material

Below is the link to the electronic supplementary material.

**Supplementary Table 1**. The incidence rate of adverse events including procedures associated with suspected infection after injection according to the intra-articular hyaluronic acid injection regimen and corticosteroid (*N* = 31, 286)*

## Data Availability

The data used in the study are potentially identifiable and are not publicly available. The raw claims datasets generated and/or analyzed during the study are not publicly available due ethical restrictions by the HIRA. All data generated or analyzed during this study are based on HIRA research data (M20210727405).
