# Correction: Association between intra-articular hyaluronic acid injections in delaying total knee arthroplasty and safety evaluation in primary knee osteoarthritis: analysis based on Health Insurance Review and Assessment Service (HIRA) claim database in Republic of Korea

**DOI:** 10.1186/s12891-024-08025-5

**Published:** 2024-11-14

**Authors:** Jun-Gu Park, Juho Sim, Seung-Beom Han

**Affiliations:** 1 Department of Orthopaedic Surgery, College of Medicine, Anam Hospital, Korea University, 73, Goryeodae-ro, Seongbuk-gu, Seoul, Republic of Korea; 2 Department of Preventive Medicine, Yonsei University College of Medicine, Seoul, Republic of Korea

**Correction: BMC Musculoskelet Disord 25**,** 706 (2024)**

10.1186/s12891-024-07698-2

Following the publication of the original article [1], the authors corrected the statement in Data availability section to mention Health Insurance Review and Assessment (HIRA) as this article uses HIRA data.

**Old data availability statement**:

All data from this study are not publicly available as they have been deposited in the National Health Insurance Service-Health Screening (NHIS-HealS) Database. However, all data generated or analyzed during this study are included in this published article.

**Corrected data availability statement**:

The data used in the study are potentially identifiable and are not publicly available. The raw claims datasets generated and/or analyzed during the study are not publicly available due ethical restrictions by the HIRA.

All data generated or analyzed during this study are based on HIRA research data (M20210727405).

The original article [1] has been updated.
