# Supplementary material for: Association between intra-articular hyaluronic acid injections in delaying total knee arthroplasty and safety evaluation in primary knee osteoarthritis: analysis based on Health Insurance Review and Assessment Service (HIRA) claim database in Republic of Korea
Source: BMC Musculoskelet Disord. 2024 Sep 4;25:706. doi: 10.1186/s12891-024-07698-2 (PMC11373335; doi:10.1186/s12891-024-07698-2)
Supplement: Supplementary file 1 — Supplementary Table 1. The incidence rate of adverse events including procedures associated with suspected infection after injection according to the intra-articular hyaluronic acid injection regimen and corticosteroid (N = 31, 286)* [file 12891_2024_7698_MOESM1_ESM.docx]

**Supplementary Table 1.** **The incidence rate of adverse events including procedures associated with suspected infection after injection according to the intra-articular hyaluronic acid** **injection regimen and corticosteroid (N=31,286)***

|  | **IA-HA** injection regimens without **CS** | | | **IA-HA** injection regimens with **CS** | **CS** without **IA-HA** |
| --- | --- | --- | --- | --- | --- |
|  | **Group Total.** | **IA-HA** single injection regimens without CS | **IA-HA** multiple injection regimens only without **CS** |  |  |
|  | (N=11,835) | (N=884) | (N=10,951) | (N=7,541) | (N=11,910) |
| **Procedures associated with suspected infection, n (%)**** | 797 (6.7%) | 32 (3.6%) | 765 (7.0%) | 1,051 (13.9%) | 1,078 (9.1%) |
| Septic arthritis (ICD-10: M009), n (%) | 12 (0.1%) | 1 (0.1%) | 11 (0.1%) | 16 (0.2%) | 36 (0.3%) |
| Arthroscopy (E7500), n (%) | 0 | 0 | 0 | 1 (0.01%) | 5 (0.04%) |
| Procedures in Knee-joint (Aspiration/arthrosis, Joint lavage), n (%) | 446 (3.8%) | 16 (1.8%) | 430 (3.9%) | 688 (9.1%) | 592 (5.0%) |
| Antibiotics (Oral, Injection), n (%) | 383 (3.2%) | 19 (2.1%) | 364 (3.3%) | 445 (5.9%) | 609 (5.1%) |

* Patients who did not received intra-articular hyaluronic acid injections were excluded from additional analysis.

**Infection cases were defined as the occurrence of any one of the following cases within 2 weeks of intra-articular hyaluronic acid or corticosteroid injection: Septic arthritis (ICD-10: M009), Arthroscopy (E7500), Procedures in Knee-joint (including aspiration, arthrosis, joint lavage), Prescription of and oral or injectable antibiotics.

IA-HA, Intra-articular Hyaluronic acid; CS, Corticosteroid
